# A Pilot Study of Short-Duration Hyperbaric Oxygen Therapy to Improve HbA1c, Leukocyte, and Serum Creatinine in Patients with Diabetic Foot Ulcer Wagner 3-4

**DOI:** 10.1155/2018/6425857

**Published:** 2018-08-12

**Authors:** Hendry Irawan, I. Nyoman Semadi, I. Gde Raka Widiana

**Affiliations:** ^1^Surgery Department, Faculty of Medicine, Udayana University, Sanglah General Hospital, Denpasar, Indonesia; ^2^Thorax and Cardiovascular Surgery Division, Surgery Department, Faculty of Medicine, Udayana University, Sanglah General Hospital, Denpasar, Indonesia; ^3^Nephrology and Hypertension Division, Internal Medicine Department, Faculty of Medicine, Udayana University, Sanglah General Hospital, Denpasar, Indonesia

## Abstract

**Objective:**

To evaluate the short-duration hyperbaric oxygen therapy (HBOT) can improve HbA1c levels, leukocyte count, and serum creatinine levels in patients with diabetic foot ulcer (DFU) Wagner 3-4.

**Methods:**

Blood samples from all DFU patients at Sanglah General Hospital, Denpasar, were taken for HbA1c, leukocyte, and serum creatinine test before debridement procedure, and the patients were then grouped into either standard therapy or standard therapy with HBOT for 10 sessions (combination therapy). At the end of therapy, all blood tests were resumed.

**Results:**

Each group consisted of 15 patients. Results of laboratory analysis before and after treatment were significant regarding decrease of HbA1c levels in standard therapy (10.98 ± 2.37 % to 9.70 ± 2.46 %; p = 0.006), HbA1c levels in combination therapy (9.42 ± 1.96 % to 7.07 ± 1.16 %; p < 0.001), and leukocyte count in combination therapy (13.97 ± 6.24 x 10^3^ cells/*μ*L to 8.84 ± 2.88 x 10^3^ cells/*μ*L; p = 0.009). The HbA1c levels at the end of therapy were significantly different between groups (p = 0.001). Serum creatinine level was decreased only in combination therapy but it was not significant. The effect size of all variables was larger in the combination therapy, but it was not significant (p > 0.05).

**Conclusion:**

The use of short-duration HBOT on DFU reduces HbA1c levels, leukocyte count, and serum creatinine levels better than standard therapy alone. This protocol would save time and effort in future HBOT implementation. This trial is registered with ClinicalTrials.gov Identifier: NCT03615755.

## 1. Introduction

Diabetes mellitus (DM) is one of the causes of public health problems which have a worldwide influence due to its high prevalence and large economic and social consequences. The International Diabetes Federation (IDF) states that more than 371 million people in the world between the ages of 20 and 79 years have DM, while Indonesia is the seventh country with the highest prevalence of DM under China, India, USA, Brazil, Russia, and Mexico [1]. Complications of DM can include renal failure, diabetic foot ulcers, and even lower limb amputations. Diabetic patients with diabetic foot ulcers (DFUs) require long-term wound care, resulting in social and economic consequences [2]. Diabetic patients are estimated to be 15-25 % with DFUs; 40-80 % of DFU patients have an infection risk [3] and 10-20 % of DFU patients require amputation [4].

The principle purpose of DFUs is wound healing. The main components of standard therapy are controlling blood sugar, antibiotic drug, ulcer debridement, wound care, offloading, and improved blood flow/revascularization [5, 6].

In addition to standard therapies, there are adjuvant therapies such as hyperbaric oxygen therapy (HBOT), maggot therapy, growth factor therapy, collagen products, bioengineered tissue, and stem cells, also used in the management of DFUs [5, 7–9]. Hyperbaric oxygen therapy is a 100 % oxygen delivery with a pressure of 2 to 3 atmosphere absolute (ATA) in the hyperbaric chamber. The mechanism of HBOT is to increase tissue oxygen levels resulting in accelerated wound healing, decreased edema, and killing anaerobic bacteria [10, 11].

Based on previous research, we want to learn more about HBOT and its role in not only accelerating wound healing as a clinical outcome, but also improving the hematological and biochemical conditions in patient with DFU. The aim of this study was to evaluate that the short-duration HBOT can improve glycohemoglobin (HbA1c) levels, leukocyte count, and serum creatinine levels in patients with DFU Wagner 3-4.

## 2. Methods

This study uses pretest and posttest control group design, to know the role of standard therapy and combination therapy (standard therapy with adjuvant HBOT) to decrease HbA1c levels, leukocyte count, and serum creatinine levels in DFU patient Wagner 3-4. All DM patients with DFU at Sanglah General Hospital, Denpasar, who meet the inclusion and exclusion criteria and willing to follow the research procedure were included in the study. All patients are signing the agreement paper after getting research explanation. All patients were briefed on the study research using HBOT. If they are willing to participate in the study and use HBOT, they were grouped to combination therapy, and if they are willing to participate in the study but do not want to use HBOT, they were grouped to standard therapy, but if they are not willing participate, then they were excluded. The study was approved by Institutional Review Board of Medical Faculty of Udayana University and Sanglah General Hospital Denpasar with the ethical number 580/UN.14.2/KEP/2016.

Thirty diabetic patients with DFU Wagner 3-4 participated in this study. Blood test was taken by all patients for HbA1c levels, leukocyte count, and serum creatinine levels before debridement, and they were then grouped for standard therapy or standard therapy with 10 sessions of HBOT. One session of HBOT uses oxygen at 2.4 ATA for 90 minutes per day at multiplace hyperbaric chamber. This therapy is given as five sessions per week, so it takes two weeks. At the end of therapy, all blood tests were performed again in both groups.

The inclusion criteria were patients who had type 2 diabetes and DFU Wagner class 3 or 4, aged over 18 years, and underwent debridement with or without toe amputation. The exclusion criteria were patients who had severe organs dysfunction such as heart failure, pulmonary infection, pneumothorax, chronic obstructive pulmonary disease, and stroke.

Statistical analysis using SPSS 17.0 (SPSS Inc., Chicago, Illinois, USA) was conducted. All variables were described before and after treatment. Analysis pretest and posttest values on both groups were performed using paired* t*-test and independent* t*-test. The statistical test results are significant if p < 0.05.

## 3. Results

All patients were treated with insulin as antidiabetic and intravenous empiric antibiotics. We used combination short-acting and long-acting insulin daily: a dose of short-acting (NovoRapid®, insulin aspart) 8 units every 8 hours and long-acting (Lantus®, insulin glargine) 10 units at night. We grouped the patients into two groups, each group consisted of 15 patients (Table 1), with the mean age of standard therapy group 56.67 ± 8.30 years and combination therapy group 50.53 ± 7.52 years. Patients with the combination therapy group experienced longer DFUs, 6.07 ± 4.13 weeks, than standard therapy group, 3.34 ± 2.09 weeks.

Analyses of HbA1c levels, leukocyte count, and serum creatinine levels were described in Tables 2, 3, and 4. Comparison of baseline HbA1c levels between groups was not different with p = 0.059, but after two weeks there was significant difference between groups, p = 0.001 (Table 2). Evaluation pre- and posttherapy of HbA1c was significant in both groups, in standard therapy group (from 10.98 ± 2.37 % to 9.70 ± 2.46 %; p = 0.006) and in combination therapy group (from 9.42 ± 1.96 % to 7.07 ± 1.16 %; p < 0.001).

Leukocyte count at baseline and the end of therapy between groups (Table 3) was not significant (p = 0.772 and p = 0.178, respectively), although leukocyte count after two weeks in combination therapy group decreased higher than in standard therapy group. Leukocyte count in combination therapy group was significantly decreased from 13.97 ± 6.24 x 10^3^ cells/*μ*L to 8.84 ± 2.88 x 10^3^ cells/*μ*L; p = 0.009. In this study, the effect size of HbA1c and leukocyte count was not significantly different. At the end of therapy, leukocyte count between debridement and debridement with amputation was not significantly different. In combination therapy, patients who did debridement had leukocyte count 8.63 ± 3.04 x 10^3^ cells/*μ*L compared with debridement with toe amputation 9.17 ± 2.84 x 10^3^ cells/*μ*L. Compared with standard therapy, the leukocyte count was higher in debridement and debridement with amputation (11.2 ± 6.30 x 10^3^ cells/*μ*L, 10.74 ± 4.62 x 10^3^ cells/*μ*L, respectively).

In Table 4, serum creatinine levels at baseline and after two weeks of were not comparable because, in standard therapy group, the patients had good renal function, whereas, in combination therapy group, they had impaired renal function. In standard therapy group, serum creatinine levels were stable, but serum creatinine levels were little decreased in combination therapy group. The effect size was not significantly decreased in serum creatinine level (p = 0.732) between groups.

## 4. Discussion

In this study, we found that HbA1c levels were significantly decreased after therapy in the standard therapy group (p = 0.006) and in the combination therapy group (p < 0.001) compared to baseline levels. The HbA1c levels at the end of therapy were significantly different between groups (p = 0.001), whereas the difference of HbA1c was greater in the combination therapy group, but statistically not significant (p = 0.072).

This is consistent with Karadurmus et al.'s study [12], DFU patients who received 30 sessions of HBOT had very significant decrease in fasting blood sugar and HbA1c after treatment with p < 0.001. In this study, the initial HbA1c levels were 9.1 ± 1.3 % and after HBOT were 8 ± 1.1% [12]. Aydin et al.'s study [13] also showed the decrease in HbA1c levels after 30 sessions of HBOT with 8.13 ± 2.13 % to 7.04 ± 1.15 %. El-Kader and Ashmawy [14] study revealed no improvement of HbA1c levels after 40 sessions of HBOT with 7.56 ± 1.35 % to 7.61 ± 1.38 %, but there was significant improvement of ulcers at the end of therapy compared with other treatment groups with p < 0.05.

The decreased of HbA1c levels in DFU patient showed the good effect of HBOT to improve the glycemic condition. The other studies evaluated blood glucose and fasting blood glucose levels as glycemic condition; there was a significant decrease in the glycemic condition of patients after HBOT.

Gupta and Sharma [15] revealed that there was a significant decrease in blood sugar levels in diabetic patients after TOH 18 sessions. It was like in Karadurmus et al.'s study [12], the reduction in fasting blood glucose levels was significant when comparing baseline with after 10 sessions of HBOT, the 10 sessions of HBOT with the 20 sessions of HBOT, and the 20 sessions of HBOT with the 30 sessions of HBOT. The decrease in blood sugar levels was 24.7% in Karadurmus et al.'s study [12] and this resembled the study of Al-Waili et al. [16], 23%.

Until now, the mechanism of glucose metabolism during use of HBOT remains unclear. The researchers found the increase in insulin receptor activity and changes of insulin sensitivity through increased PPAR-*γ* (peroxisome proliferator-activated receptor-*γ*) regulation. It can decrease blood sugar levels without increased pancreatic insulin secretion [17]. In addition, insulin sensitivity also increased within 3 days after HBOT up to 30 sessions of HBOT [18].

In this study, the leukocyte count was decreased at the end of therapy in both groups, but only in the combination therapy group it had a significant decrease (p = 0.009) compared to baseline. At the end of therapy, there was decrease of leukocyte count higher in combination therapy group. But the difference between group was statistically not significant with p = 0.468.

The result of our study was the same as that of Karadurmus et al.'s study [12], DFU patients after 30 sessions of HBOT had a significant reduction of leukocyte count, from 11.2 ± 3.0 x 10^3^ cells/*μ*L to 7.7 ± 2.1 x 10^3^ cells/*μ*L; p < 0.001. That study also evaluated that CRP (C-reactive protein) as inflammatory marker was significantly decreased (p < 0.001) after administering HBOT [12]. Decreasing of inflammatory marker showed that HBOT can be used as a bactericide and decrease in inflammatory cytokines occurred in DFUs [12, 19]. Leukocytes fight infections in the ulcer using 20 times more oxygen when killing bacteria [14].

However, in Gupta and Sharma's study [15], there was an increase of leukocyte count in diabetic patients after 18 sessions of HBOT, but there was significant decrease of neutrophils after HBOT. El-Kader and Ashmawy [14] research showed that an inflammatory marker of CRP was higher in DFU patients after receiving 40 sessions of HBOT.

On the condition of breathing with oxygen, more than 1 ATA will increase reactive oxygen species (ROS) which plays a role in redox reactions, cell signaling, and antioxidant [20]. Besides that, the enhancement of ROS plays a role in healing or neovascularizing the wound and improving tissue after ischemic conditions. This condition is characterized by increased variety of growth factors, stimulating the proliferation and migration of cells, increased fibroblasts, increased cytokines, increased angiogenesis and neovascularization, and increased synthesis of extracellular matrix [19–21].

Hyperbaric oxygen therapy also increases the formation of oxygen free radicals, which oxidize proteins and membrane lipids, damaging the DNA (deoxyribonucleic acid) of bacteria, and inhibit bacterial metabolic functions. A previous study found an increase in polymorphonucleocytes and macrophages as bacteriocidal effect when the oxygen pressure in the infected tissue is high. Hyperoxia during HBOT will inhibit toxin production of clostridia and increase the potential of antibiotics such as fluoroquinolones, amphotericin B, and aminoglycosides, which use oxygen for transport across the cell membrane [11, 19, 22, 23]. However, infection in diabetic foot ulcers will worsen if glycemic control is poor [24].

In this study, serum creatinine levels were not comparable between groups, as standard therapy group had low serum creatinine levels, whereas the combination therapy group had high serum creatinine levels. In the combination therapy group, the serum creatinine level decreased slightly from 2.1 ± 2.88 mg/dL to 2.05 ± 2.77 mg/dL; p = 0.551, whereas in the standard therapy group the serum creatinine level was stable.

In Fife et al.'s study [25], using HBOT in renal failure patients, 79 of 136 (58%) had improvement of renal function and, in patients with no renal failure, 638 of 835 (76%) had improvement of renal function, with p < 0.00001. Patients experienced improvement after mean using 34 sessions of HBOT, but mean using 24 sessions of HBOT did not improve [25]. In Kevin's study [26], 5 sessions of HBOT did not affect glomerular filtration rate values before and after therapy (p = 0.097) in DFUs.

Ayvaz et al.'s study [27], using rats with acute renal failure model, in 2 sessions of HBOT group had a significantly higher decrease in serum urea and creatinine levels (p < 0.005) than the non-HBOT group. In addition, histopathological examination showed that HBOT group decreased necrosis cells, decreased cylinder/cast formation, and decreased apoptotic cells [28]. This was similar to Solmazgul et al. [29], using rats model of renal reperfusion/ischemia, the assessment of renal function with serum urea and serum creatinine levels was significantly higher than in healthy rats (p < 0.05). In groups of rats undergoing renal reperfusion/ischemia given HBOT, there was a significant decrease in serum urea and creatinine levels (p < 0.05) compared to the non-HBOT group. The HBOT effects can reduce the occurrence of damage to renal tissue and reduce infiltration neutrophils that play an important role in renal injury [29]. In Migita et al. [28], the effect of HBOT may decrease apoptotic cells by increasing the proliferative reaction after renal reperfusion/ischemia in rats.

Rubinstein et al.'s study [30] showed an improvement in glomerular filtration rate in the group who received 2 sessions of HBOT in 24 hours after renal ischemia, at 2 hours and 22 hours. In renal ischemia without HBOT, there was decrease in glomerular filtration rate of 94% compared to healthy renal, and in renal ischemia with HBOT there was decrease in glomerular filtration rate of 68% compared to healthy renal with significant difference (p < 0.05). In this study, HBOT improved renal vasodilation associated with increased blood flow to the renal, thus improving renal cortical perfusion [30]. Berkovitch et al.'s study [31], using healthy rats given HBOT did not cause impaired renal function, which was assessed with normal serum urea and serum creatinine levels [31].

In conditions of hyperglycemia, glycation of various proteins occurs and collagen formed advanced glycation end-products (AGEs). This will lead to damage to the body, such as vascular disorders, platelet activation and aggregation, increased free radicals and inflammation, and immune system impairment. Increased AGEs will lead to complications of DM patients such as nephropathy [32].

Evaluation of the short-duration HBOT on improving renal function in humans has not shown significant results, including in our study. However, in animal experiments, HBOT has an effect of improving renal function. Further research can be done to evaluate influence of HBOT in DFU patients with larger sample, control group uniformity and treatment, and other assessed variables, including biomolecular markers.

## 5. Conclusion

Hyperbaric oxygen therapy of 2.4 ATA for 90 minutes per day for 10 sessions has the benefit of decreasing HbA1c levels and leukocyte count of patients with DFU Wagner 3-4. However, there is little effect of HBOT on serum creatinine levels as a sign of improved kidney function. This shows that HBOT can be used as a therapy that helps standard therapy in handling DFU in reducing glycemic and inflammatory levels. The use of short-duration HBOT on DFU would save time and effort in future HBOT implementation.

## Figures and Tables

**Table 1:** Characteristics of patients.

**Variables**	**Standard therapy**	**Standard therapy + HBOT**
	**(n=15)**	**(n=15)**
Age (years old)^a^	56.67 ± 8.30	50.53 ± 7.52
Duration of DFU (weeks)^a^	3.34 ± 2.09	6.07 ± 4.13
Duration of DM (years)^b^	4 (1-10)	3 (0.17-25)
Body mass index (kg/m^2^)^b^	22.04 (14.33-36.73)	22.04 (17.58-29.40)
Sex (%)		
Male	4 (26.7)	8 (53.3)
Female	11 (73.3)	7 (46.7)
Smoking (%)		
Yes	1 (6.7)	3 (20)
No	14 (93.3)	12 (80)
Hypertension (%)		
Yes	6 (40)	6 (40)
No	9 (60)	9 (60)
Ulcer classification (%)		
Wagner 3	8 (53.3)	6 (40)
Wagner 4	7 (46.7)	9 (60)
Surgery (%)		
Debridement	9 (60)	9 (60)
Debridement with toe amputation	6 (40)	6 (40)
Hemoglobin (g/dL)^a^		
Baseline	10.47 ± 1.72	10.34 ± 1.55
After 2 weeks	9.45 ± 1.46	11.04 ± 1.47
Albumin (g/dL)^a^		
Baseline	3.00 ± 0.56	3.08 ± 0.78
After 2 weeks	3.07 ± 0.53	3.59 ± 0.76

^a^mean ± standard deviation; ^b^median (range).

**Table 2:** HbA1c levels between groups.

**HbA1c (**%**)**	**Standard therapy**	**95**%** CI**		**Combination therapy**	**95**%** CI**		**p**
		**Lower**	**Upper**		**Lower**	**Upper**	
Baseline	10.98 ± 2.37	9.67	12.30	9.42 ± 1.96	8.33	10.50	0.059
After 2 weeks	9.70 ± 2.46	8.34	11.06	7.07 ± 1.16	6.43	7.72	0.001^*∗*^
Effect size	1.28 ± 1.54	0.43	2.13	2.34 ± 1.57	1.47	3.21	0.072

**p**	0.006^*∗*^			< 0.001^*∗*^			

All values were mean ± standard deviation; ^*∗*^p value < 0.05.

**Table 3:** Leukocyte count between groups.

**Leukocyte (10** ^**3**^ ** cells/** **µ** **L)**	**Standard therapy**	**95**%** CI**		**Combination therapy**	**95**%** CI**		**p**
		**Lower**	**Upper**		**Lower**	**Upper**	
Baseline	14.27 ± 6.79	10.51	18.03	13.97 ± 6.24	10.51	17.43	0.772
After 2 weeks	11.01 ± 5.51	7.96	14.07	8.84 ± 2.88	7.26	10.43	0.178
Effect size	3.26 ± 7.76	-1.04	7.56	5.13 ± 6.72	1.40	8.85	0.468

**p**	0.14			0.009^*∗*^			

All values were mean ± standard deviation; ^*∗*^p value < 0.05.

**Table 4:** Serum creatinine levels between groups.

**Serum creatinine (mg/dL)**	**Standard therapy**	**95**%** CI**		**Combination therapy**	**95**%** CI**		**p**
		**Lower**	**Upper**		**Lower**	**Upper**	
Baseline	0.73 ± 0.27	0.58	0.89	2.10 ± 2.88	0.50	3.69	
After 2 weeks	0.73 ± 0.27	0.58	0.89	2.05 ± 2.77	0.52	3.59	
Effect size	0 ± 0.13	-0.07	0.07	0.05 ± 0.45	-0.21	0.29	0.732

**p**	0.985			0.551			

All values were mean ± standard deviation.

## Data Availability

The data used to support the findings of this study are available from the corresponding author upon request.
